# Acceptance of Mobile Health in Communities Underrepresented in Biomedical Research: Barriers and Ethical Considerations for Scientists

**DOI:** 10.2196/mhealth.6494

**Published:** 2017-06-28

**Authors:** Camille Nebeker, Kate Murray, Christina Holub, Jessica Haughton, Elva M Arredondo

**Affiliations:** ^1^ Center for Wireless and Population Health Systems Qualcomm Institute University of California, San Diego La Jolla, CA United States; ^2^ Scripps Translational Science Institute Scripps Health La Jolla, CA United States; ^3^ Department of Family Medicine and Public Health School of Medicine University of California, San Diego La Jolla, CA United States; ^4^ School of Psychology and Counselling Queensland University of Technology Queensland Australia; ^5^ Department of Public Health California State University San Marcos San Marcos, CA United States; ^6^ Institute of Behavioral and Community Health Graduate School of Public Health San Diego State University San Diego, CA United States

**Keywords:** telemedicine, cultural diversity, ethics, research, ethics committees, research, privacy, informed consent

## Abstract

**Background:**

The rapid expansion of direct-to-consumer wearable fitness products (eg, Flex 2, Fitbit) and research-grade sensors (eg, SenseCam, Microsoft Research; activPAL, PAL Technologies) coincides with new opportunities for biomedical and behavioral researchers. Underserved communities report among the highest rates of chronic disease and could benefit from mobile technologies designed to facilitate awareness of health behaviors. However, new and nuanced ethical issues are introduced with new technologies, which are challenging both institutional review boards (IRBs) and researchers alike. Given the potential benefits of such technologies, ethical and regulatory concerns must be carefully considered.

**Objective:**

Our aim was to understand potential barriers to using wearable sensors among members of Latino, Somali and Native Hawaiian Pacific Islander (NHPI) communities. These ethnic groups report high rates of disparate health conditions and could benefit from wearable technologies that translate the connection between physical activity and desired health outcomes. Moreover, these groups are traditionally under-represented in biomedical research.

**Methods:**

We independently conducted formative research with individuals from southern California, who identified as Latino, Somali, or Native Hawaiian Pacific Islander (NHPI). Data collection methods included survey (NHPI), interview (Latino), and focus group (Somali) with analysis focusing on cross-cutting themes.

**Results:**

The results pointed to gaps in informed consent, challenges to data management (ie, participant privacy, data confidentiality, and data sharing conventions), social implications (ie, unwanted attention), and legal risks (ie, potential deportation).

**Conclusions:**

Results shed light on concerns that may escalate the digital divide. Recommendations include suggestions for researchers and IRBs to collaborate with a goal of developing meaningful and ethical practices that are responsive to diverse research participants who can benefit from technology-enabled research methods.

**Trial Registration:**

ClinicalTrials.gov NCT02505165; https://clinicaltrials.gov/ct2/show/NCT02505165 (Archived by WebCite at http://www.Webcitation.org/6r9ZSUgoT)

## Introduction

We have rapidly entered an era where personal health data (PHD) is collected on-the-fly and in real time, which is vastly expanding our ability to design and test personalized and adaptive health interventions [1]. Direct-to-consumer fitness products (eg, Fitbit, MapMyFitness) and wearable research tools (eg, SenseCam, ActivPAL) offer great potential for tracking of PHD and may serve as catalysts for behavior change within communities where health disparities are most prevalent. There are persistent health disparities in the United States, with numerous currently underserved communities who could benefit from mobile technologies designed to facilitate awareness and change in health behaviors [2].

Despite this great opportunity, a recent study evaluating the use of health apps revealed that disparities persist among racial and ethnic minority groups who are non-native English speakers and have lower levels of educational attainment [3]. A systematic review of health-related technology use by “historically underserved health consumers” revealed little progress on the development or use of culturally-informed technologies designed to reduce health disparities [2]. If adoption of health technology is a national priority [2], we clearly have a gap to fill to address disparities in technology use. Whereas barriers to engaging diverse communities with research have been documented [4], there is little information guiding researchers on barriers specific to the use of health-related technologies across diverse populations. In addition to the issues of access and equity, research studies that collect PHD using wearable sensing technologies are raising new and nuanced ethical challenges that also require attention [5].

Researchers who are using mobile health (mHealth) methods and tools can remotely record a variety of individual level data, including the participant’s location, physiology, mood, and social interactions. For example, researchers can now objectively measure sedentary behavior using a wearable accelerometer sensor [6], stimulate autobiographical memory with a wearable camera [7], monitor mental health with smartphone capabilities [8], mine social media to predict disease outbreaks [9], and track geographic location to contextualize health behaviors [10] **.** Although the potential is exciting, researchers and institutional review boards (IRBs) are independently questioning the new ethical challenges introduced by this research (ie, informed consent, bystander rights) [5].

According to the US Census, California is considered a minority-majority state, with no single ethnic group forming a majority of the state’s population. Within southern California and, specifically San Diego County, public health research and service initiatives are actively addressing health disparities within ethnically diverse communities. To explore interest in using mobile and wearable technologies for health research purposes, three independent formative pilot studies were initiated that focused on Latino, Somali, and Native Hawaiian Pacific Islander (NHPI) communities in southern California. This commentary brings together lessons learned from these pilot studies and reports the ethical, legal, and social implications raised by a sample of culturally diverse community members; stakeholders often neglected in discussions to inform ethical research practices. We applied lessons learned in the form of recommendations for scientists interested in using digital technologies with culturally diverse communities and to IRBs charged with protecting human subjects.

### Pilot Studies

To identify potential barriers and motivators to participating in mHealth studies, the authors independently queried a sample of culturally diverse community members to identify perspectives about wearable and wireless sensing technologies. These independent inquiries were not coordinated in advance and, as such, different methodologies were utilized. The samples included Latino women, Somali women, and men and women from NHPI communities, each of whom experienced health disparities and might benefit from wearable technologies that translate the connection between physical activity and desired health outcomes.

### Pilot Methods

Researchers working within the Latino community (JH, EA) conducted individual interviews with 10 Latino women to learn whether they would be willing to wear a global positioning system (GPS) location-tracking device as part of a health promotion study. These individuals were recruited from a larger sample of women already participating in the *Fe en Acción* study [11] and who consented to be contacted for further studies. The interviewer was bilingual/bicultural and worked as a research assistant on the larger study. Another researcher (KM) conducted a focus group with 5 adult Somali women who participated in a pilot study of a culturally adapted physical activity program [12] where compliance with wearing an activity monitor was low. These women were part of an initial pilot of the program to be tested using a randomized controlled trial design. To gauge barriers and motivators to wearing a GPS and activity monitors among the NHPI community, researcher (CH) surveyed 39 participants. The survey was self-administered, using paper and pen, to participants who were recruited from social, civic, and other cultural organizations. Each study was conducted under an IRB-approved protocol. Table 1 provides information about each of the studies and citations for further detail, where available. The purpose of this commentary is to highlight general findings of barriers to the adoption of mHealth research tools for communities currently underrepresented in research. A summary of lessons learned follows, to promote discussion in the field and to guide future research initiatives.

**Table 1:** Review of three pilot study samples, methodologies, and findings.

Study population	Facilitation	Informed consent	Format (type of data)	Privacy and confidentiality	Data collection and technology assessed	Key findings
Adult Latino women [11] (n=10) mean age: 49.3 years	In Spanish by research assistant	Written and verbal consent	Interviews (qualitative)	Names replaced with IDs; transcripts kept confidential	Interviews conducted following 12-month intervention regarding barriers to wearing an accelerometer and GPS^a^ device	Unfamiliar with GPS technology Concerns about device safety Misconceptions about data collected
Adult Somali women [12] (n=5) mean age: 46.1 years	In Somali by bicultural researchers	Written and verbal consent	Focus groups (qualitative)	Written notes without names; group confidentiality discussed	Focus group at end of 6-week intervention trial regarding lack of compliance with wrist-worn accelerometer	Unfamiliar with accelerometer and data gathered Unwanted attention Inconvenient
Adult Pacific Islanders (n=39) mean age: 38.0 years	In English by research assistant	Verbal consent only	Self-administered survey (quantitative)	Privacy and confidentiality discussed verbally; study IDs used, no names	Survey items included barriers to wearing an accelerometer and GPS device	Concerns about privacy and data access Concerns about being tracked

^a^GPS: global positioning system.

**Pilot Findings**

Researchers who interviewed Latino participants reported a lack of familiarity with the location-tracking technology, misconceptions about what would be tracked, and difficulty understanding the concept of measurement in “real time.” Participants also expressed concerns about device safety and perceived an elevated risk to those lacking legal documentation to be in the United States [13]. There were examples of misconceptions about safety, with one participant stating:

Depending on the device, which could cause something, maybe like radiation or something.

Other participants were concerned about potential legal risks, with statements such as:

They have a bit of paranoia that the government always wants to know where they are, the illegals. Some people would think that (GPS) is a way to find them.

These concerns about safety and the use of data were significant barriers within the Latino sample.

During the planning phase of a community-based participatory research (CBPR) study conducted within the local Somali community [12], the research team reported low compliance among participants who had agreed to use a wrist-worn accelerometer. A post-pilot study focus group was convened to explore participant experiences and assess the appropriateness of the study design. Results identified that participants were unfamiliar with the wearable technology and uncertain about the type and quantity of data collected. Participants revealed that the device prompted questions from others (unwanted attention) and was inconvenient to wear, as the ritual of prayer in the Muslim community is observed five times per day and require that one wash prior to praying. This inconvenience prompted participants to ask if the accelerometer could be worn on a belt around their waist to decrease inconvenience and unwanted attention. Likewise, participants indicated that they wanted study information to share with family and friends who were curious about the device and their participation.

Those working with the local NHPI community in a CBPR focusing on physical activity and sedentariness surveyed 39 participants who were involved in formative research to inform the research plan. Specific to the wearable technologies, participants were concerned about wearing a location-tracking device, citing interference with lifestyle and worries related to privacy and data confidentiality. When asked about the accelerometer, participants questioned who would have access to their information, as well as how their information would be shared and reported. Participants repeated expressions related to privacy and surveillance such as “I like to keep my affairs private...who is tracking (me)?” and “I don't like knowing that I’m being tracked.” 

This was further supported by participants from the *Fe en Acción* study who stated:

I think that it invades privacy a bit. For some people, I think there is more danger than for others.

The concerns around privacy and the potential risks related to such data were consistent across all groups.

### Ethical Principles

These summaries introduce potential barriers that may perpetuate disparities and decrease access to prevention research targeting communities where health disparities are more prevalent. Responses from participants representing these three distinct communities point to potential challenges explaining the technology (eg, informed consent), data management (eg, participant privacy, data confidentiality, data sharing conventions), social implications (eg, unwanted attention), and legal risks (eg, undocumented status).

These challenges also align with the guiding ethical principles of autonomy, beneficence, and justice described in the Belmont Report [14]. Specifically, steps to decrease barriers may involve leveraging the three principles in this ethical framework when designing studies using mHealth tools and/or methods. For example: (1) Autonomy or respect for persons is demonstrated by obtaining meaningful informed consent and recognizing that several approaches (eg, visual, bullet points) may be necessary when communicating complex study information with individuals who may not be technology-savvy consumers; (2) Beneficence involves weighing risk and benefit in an era of seemingly limitless data collection with increased sensitivities to privacy, data confidentiality, and culture; and, (3) Justice focuses on decreasing inequities in access to technology and research through education and stakeholder engagement. Although these challenges are not unique to culturally diverse communities, the three groups represented are currently underrepresented in research and efforts should be made to increase access.

### Implications for Scientists and Research Ethics Boards

Using the principles of the Belmont Report as a framework, we lay down recommendations to reduce barriers to participate in research studies that use pervasive sensing methods and tools to collect personal health data.

#### Autonomy: Informed Consent

The informed consent process is a cornerstone to ensuring an individual’s right to autonomy is upheld and is a key element of the principle of respect [14]. Demonstrating autonomy requires that people participate voluntarily after receiving “adequate” information about the research. In practice, communicating complex information to people who may be unfamiliar with the scientific method, the technologies utilized, and the data produced poses considerable challenges to obtaining informed consent. Numerous studies have shown that the traditional method of conveying complex concepts via a written document is not effective, even when the consent language is simplified [15].

In line with current recommendations to reduce health disparities [16], we recommend that researchers engage with community members during the research design process to learn about barriers and motivators to the use of passive wearable sensing technologies to collect PHD. Likewise, efforts should be made to educate individuals who may become research participants to improve their ability to make informed decisions in studies that employ pervasive sensing strategies. Creating a meaningful informed consent process is critical and will likely require involving participants as partners who are willing to review and modify consent language and processes to increase access and understanding. Furthermore, education about technologies used in research can reduce barriers associated with a lack of familiarity and, subsequently, increase trustworthiness of the research enterprise. We recommend formative research be carried out with representatives of underserved communities to explore, for example:

- the acceptability of current practices for obtaining informed consent,
- how best to communicate complex concepts related to technology and data,

- preferences for privacy and data security, and
- how learning styles and literacy levels influence consent comprehension.

Data from this formative research can then be used to support alternatives to the traditional informed consent content and processes. Designing a meaningful informed consent process also requires that IRBs be willing to consider alternatives to the institutional templates that do more to protect institutions than facilitating an informed participant. These alternatives may involve experimenting with (1) less complex content (ie, legalese), (2) a tiered information presentation structure beginning with straightforward bullet points, and (3) conceptualizing the consent process as an opportunity to develop a relationship with a prospective participant rather than for documenting a transaction.

#### Beneficence: Weighing Risk and Benefit

The principle of beneficence is demonstrated by evaluating the probability and magnitude of harm in relation to the potential benefits of the research to an individual and people to whom the results may be generalized. There is little empirical evidence to guide IRB risk assessment, including threats to participant privacy if the data are breached and proper security practices to protect the amount of data collected using these methods. Pervasive sensing methods capture vast quantities of granular private identifiable information and personal health data—much of which is not protected by regulations that cover patient electronic health records. In addition, visual, audio, and location-tracking sensors may pick up information about people who are in close proximity to a research participant. These people, whom we call a “bystander” or a “by-catch,” do not meet the definition of a human subject and, therefore, their rights may not be considered by an IRB. Yet, these individuals may expect to grant permission if recorded by a research participant. This concern about “tracking” a person who is with the research participant was raised by a Latino participant who believed that a GPS may introduce a potential legal risk for undocumented individuals who travel with the participant. This sensitivity may be magnified where legal matters, such as immigrant status, are concerned.

In an era of limitless opportunities to collect information, thoughtful discussions should guide what is and what is not collected to ensure maximum benefit to participants and science. As noted, participants in these three formative pilot studies expressed concerns around device safety (ie, GPS), data management (ie, handling of confidential and personal information), and potential legal risks. We recommend that standards for securely storing the volume of PHD generated via these new methods be developed and vetted by data security experts to reduce the risk for a data breach. Likewise, if the rights of a bystander are to be considered during the ethical review process (eg, when capturing data of individuals who have not directly provided informed consent), standardized protocols are needed to guide responsible practices.

#### Justice: Inequities in Access and Utilization

Unequal access to research and interventions utilizing pervasive sensing technologies underscores ethical challenges to principles of justice. This principle is demonstrated by making sure people included in the research represent those who will ultimately benefit from scientific findings. As with clinical research and interventions more broadly [17,18], better tracking and accountability efforts are needed to improve recruitment and retention of diverse samples. To advance these efforts, we believe researchers and funding agencies have responsibilities and recommend more systematic tracking of critical factors such as language preference, country of origin, health literacy, and socioeconomic status at screening and enrollment to identify points at which underserved communities are selected out of trials and studies. While disparities in research participation are noted [17,18], there is currently limited data to support when and why there are biases in recruitment and retention. More systematic reporting on these factors would allow for greater understanding and direct efforts to ensure greater representation. Greater support of formative research, such as those described here, are needed to identify ways to reduce barriers at identified points of attrition and to hold studies accountable for their ability to recruit and retain samples that mirror the general population.

There are a few limitations worth considering. Because there were no majority group comparisons, we were unable to comment on how the challenges in implementing mHealth studies encountered by our participants compare to those with members of majority groups that may explain the digital divide. Furthermore, our participants were recruited using convenience sampling, which limits our ability to generalize to the target groups. In addition, the methodologies used across these studies were not the same, which makes it difficult to make direct comparisons and conduct more in-depth analyses. More nuanced studies are needed to tease apart the relative weight of cultural, linguistic, and educational differences across different communities and subgroups that may vary in acculturation and exposure to wearable sensor technologies used in mHealth research.

## Conclusions and Next Steps

The growth of research using wearable and passive sensing technologies provides a tremendous opportunity to overcome linguistic and literacy barriers to engaging currently underserved communities in public health research and interventions. Thoughtful steps are needed to ensure equal access, or else there will be a significant danger of perpetuating or even escalating current disparities. Our commentary sheds light on concerns that may escalate the digital divide and provides suggestions for how scientists can mitigate barriers when working with underserved and culturally diverse communities. Moving forward, we suggest that mHealth researchers and IRBs work together to create meaningful ethical research practices that are responsive to research participants and consumers who can benefit from research in the digital age.

## Abbreviations

CBPR: community-based participatory research
GPS: global positioning system
IRBs: institutional review boards
mHealth: mobile heath
NHPI: Native Hawaiian Pacific Islander
PHD: personal health data
